# Visiting List

**Published:** 1882-02

**Authors:** 


PHYSICIAN'S VISITING LIST AND PRESCRIPTION RECORD. PERPETUAL. Otis Clapp & Son, Boston and Providence. 1882.

A very neat book; useful to those who keep no other record of their cases than the name of the medicine given. It will be of service, however, to those who keep a full record of their case, for noting charges, visits, etc. It differs from others in being "perpetual."

